# Fatal Manifestations of Methotrexate Overdose in Case of Psoriasis Due to Dosing Error

**DOI:** 10.7759/cureus.30041

**Published:** 2022-10-07

**Authors:** Shubham V Nimkar, Pallavi Yelne, Shilpa A Gaidhane, Sourya Acharya, Sunil Kumar

**Affiliations:** 1 Department of Medicine, Jawaharlal Nehru Medical College, Datta Meghe Institute of Medical Science (Deemed to be University), Wardha, IND; 2 School of Epidemiology and Public Health, Jawaharlal Nehru Medical College, Datta Meghe Institute of Medical Sciences (Deemed to be University), Wardha, IND; 3 Department of Medicine, Jawaharlal Nehru Medical College, Datta Meghe Institute of Medical Sciences (Deemed to be University), Wardha, IND

**Keywords:** dosing error, fatal, psoriasis, methotrexate toxicity, critical care

## Abstract

For more than 50 years, methotrexate (MTX), when used in low doses, has been one of the most commonly used drugs in the treatment of psoriasis. It has immunosuppressive as well as anti-inflammatory properties. Pancytopenia, hepatic dysfunction, respiratory toxicity, and acute renal failure are some of the reported side effects. This is a case study of a patient who had developed florid symptoms of methotrexate toxicity as a result of erroneous overdosing of methotrexate, which was given to her for psoriasis management.

## Introduction

Methotrexate (MTX) inhibits DNA synthesis, and it also inhibits repair and cellular application by antagonizing folic acid, which is required for the deoxyribonucleic acid synthesis of cells. It inhibits dihydrofolate reductase, an enzyme that converts dihydrofolate to tetrahydrofolate once within the cell. MTX has anti-inflammatory and immunosuppressive properties when used in small doses. Low-dose MTX has been used for more than 50 years as a safe and effective treatment for psoriasis [1]. The primary route of elimination is renal excretion, which is influenced by the dosage and route of administration [2].

This case is being reported because of the unique circumstances under which the patient suffered a methotrexate overdose. This is a case study of a patient who had developed florid symptoms of methotrexate toxicity as a result of erroneous overdosing of methotrexate by a relative, which was given to her for treatment of psoriasis by the dermatologist.

## Case presentation

A 65-year-old female homemaker with a past medical history of psoriasis for 10 years was taken to the emergency department by her son with a complaint of increased skin lesions all over the body with bleeding from the lesions along with painful mouth ulcers for the last 15 days. She had been complaining of a high-grade fever associated with shivering for the last three days. Relatives had also noticed that she had been passing black-colored stools for the last two days. She had stopped taking food due to painful ulceration in her mouth.

**Figure 1:**
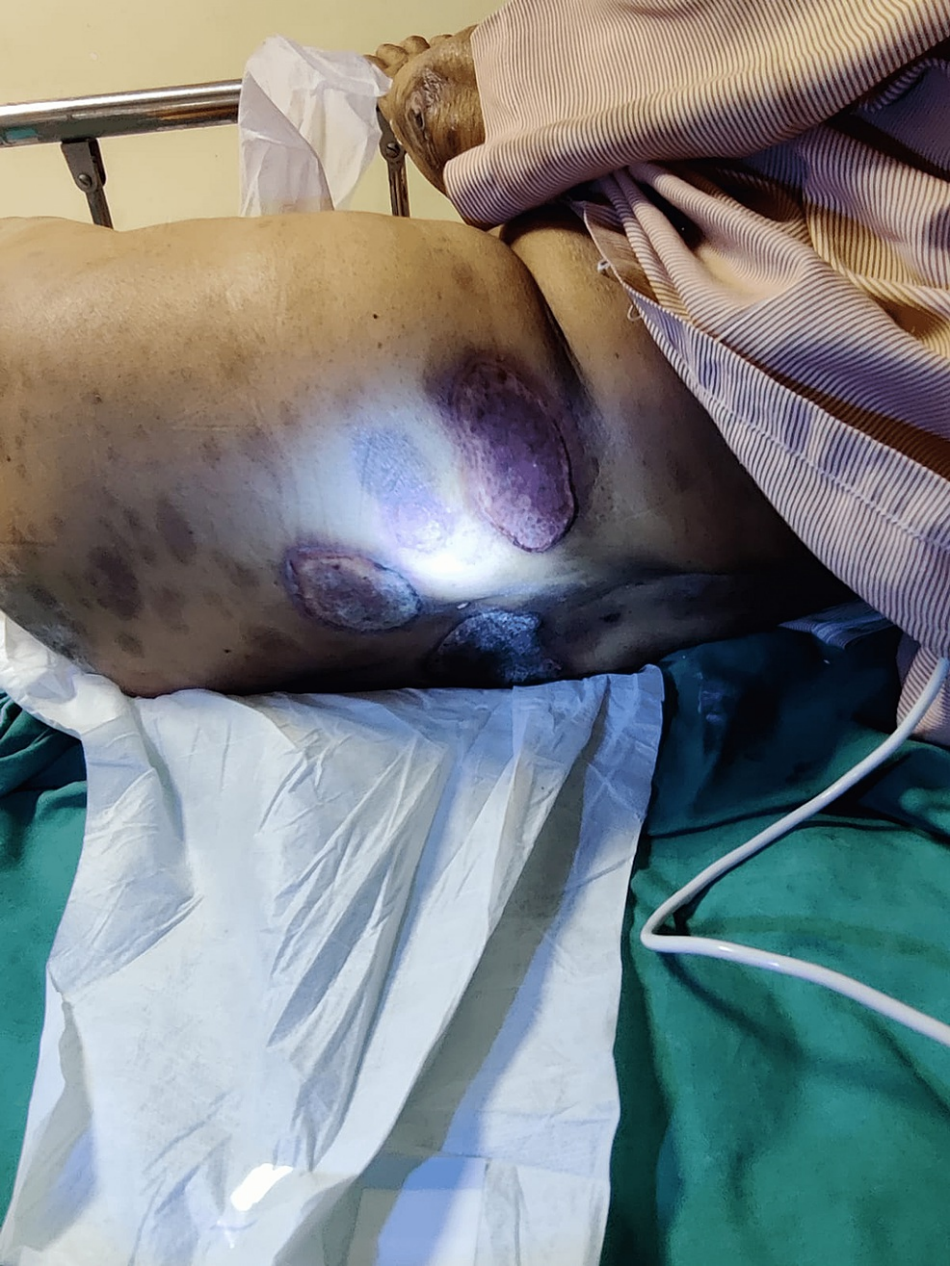
Condition 1: suggestive of hemorrhagic patches on various parts of the body

**Figure 2:**
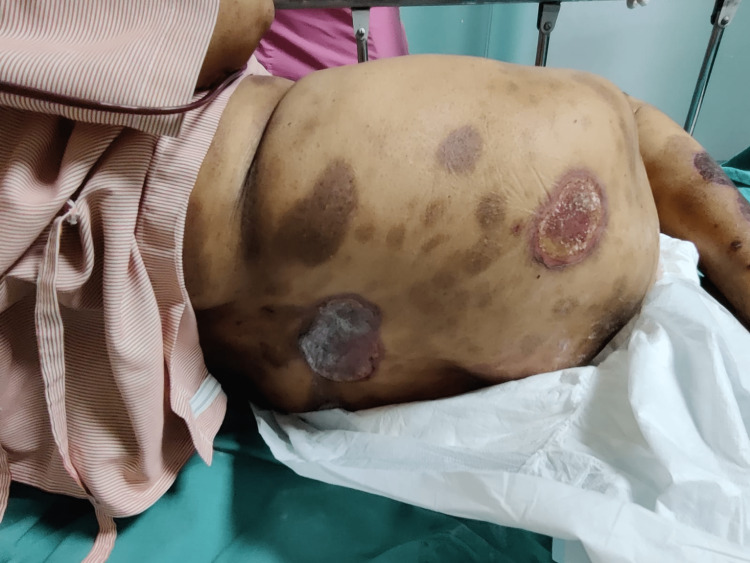
Condition 2: suggestive of hemorrhagic patches on various parts of the body

**Figure 3:**
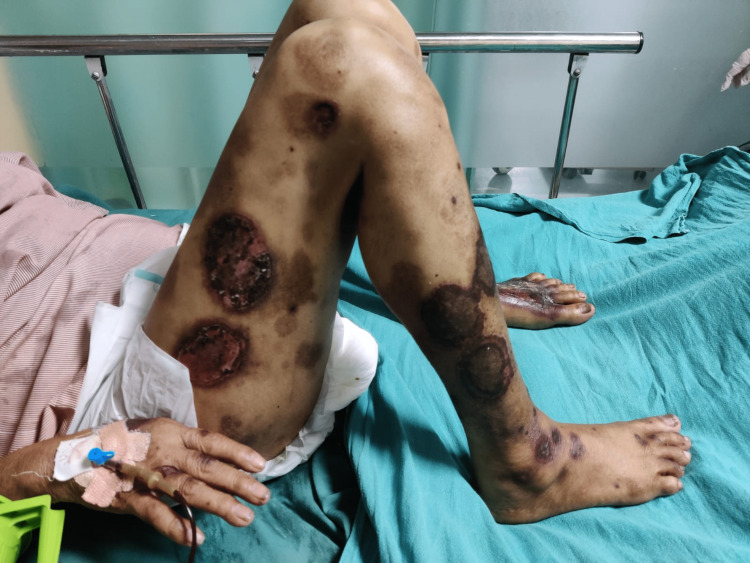
Condition 3: suggestive of hemorrhagic patches on various parts of the body

**Figure 4:**
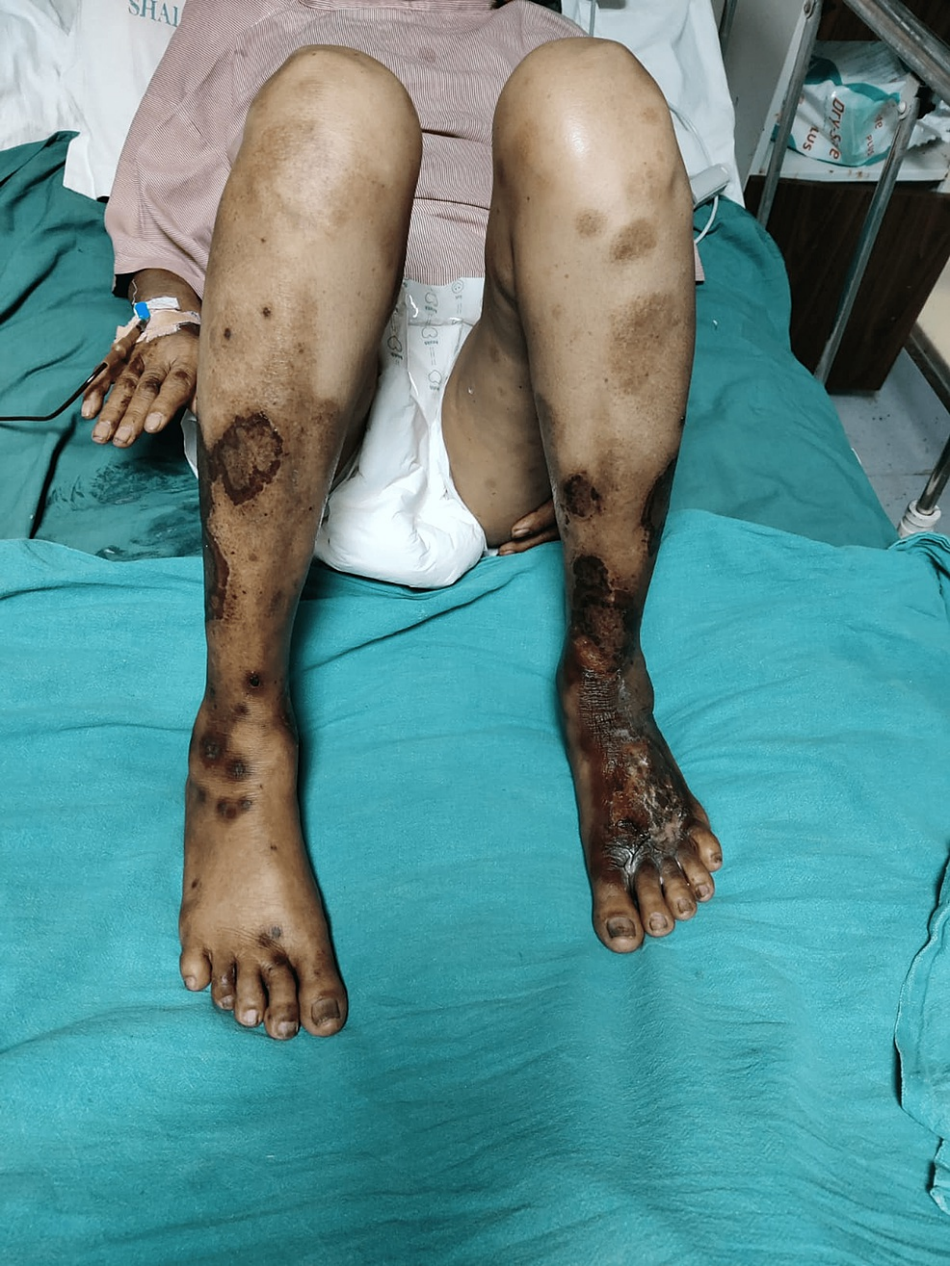
Condition 4: suggestive of hemorrhagic patches on various parts of the body

On physical examination, she was alert and oriented, afebrile with a heart rate of 88/min and regular rhythm with a blood pressure of 130/70 mm hg, RR-16/min, and pallor was present. Icterus, clubbing, and cyanosis were absent. Her systemic examination was within normal limits. However, on local skin examination, the patient had ulcerated and necrotic psoriatic plaques with redness and tenderness on various parts of the body (Figures 1-4), especially the forearm, legs, and buttock area. Signs of active bleeding from the skin lesions were present. There was blood in the stool. There was no sublingual bleed, oral bleed, or blood in the urine.

The patient was a known case of psoriasis and had been taking tab methotrexate 7.5 mg once a week and tab folic acid 5 mg once a day for 10 years. However, for the last 15 days, she was accidentally given a dose of methotrexate 7.5 mg once daily and folic acid 5 mg once a week by her relative, following which she started to manifest the toxicity signs.

Her blood investigations revealed hemoglobin of 7.7 g%, leukocyte count 100/mm^3,^ platelets were 48,000/mm^3^, and a peripheral blood smear was normal. Her blood urea was 81 mg/dl, serum creatinine was 1.2 mg/dl, serum bilirubin was 0.6 mg/dl, ALT - 27 IU/L, AST - 25 IU/L, coagulation parameters were deranged, and urine examination showed plenty of pus cells.

The patient's treatment started with injectable folinic acid 15 mg qid and the plan was for 10 doses to be given and inj filgrastim 300 µg subcutaneously once a day. Also, higher antibiotics and other supportive management were started. The patient was put in an isolated area of the medical intensive care unit. In view of low hemoglobin, low platelet count, and a deranged coagulation profile, the patient was transfused with 20 units of random donor platelets, 4 points of fresh frozen plasma, and 2 units of whole blood. Daily monitoring of platelet count as well as bleeding tendencies and hemorrhagic patches were done.

In view of the fall in oxygen saturation and a Glasgow coma scale of 6, the patient was intubated and kept on a mechanical ventilator. The patient was noted to be hypotensive with a blood pressure of 80/60 mmHg because of which she was started on inotropic support. The patient continued to deteriorate and passed away, with the cause of death being thrombocytopenia due to methotrexate toxicity.

As an exact protocol for managing methotrexate poisoning is not available, we decided to form a treatment strategy based on previously published case reports and considering the limitations of rural setup, unavailability of hematologists during emergency hours, and adequate unavailability of single donor platelets. This case is special because it happened not due to a wrong prescription by the doctor but because of an accidental overdosage by the relatives.

## Discussion

Toxicity from low-dose MTX in psoriasis is uncommon, and most cases are due to failure to follow the prescribed recommendations [1,2]. If additional methotrexate is given earlier than the normal weekly dosage, the risk of toxicity increases [3]. The self-administration of the larger, sequential dosage was the precipitating cause in our case. Not only the skin but also the gastrointestinal mucosa, liver, kidneys, and bone marrow are all affected by MTX toxicity. Since hyperproliferative psoriatic plaques absorb more methotrexate than average skin, skin ulcerations caused by MTX toxicity are limited to the psoriatic plaques [4]. This patient had ulceration on psoriasis plaques that had already formed. Patients with renal dysfunction, inflammation, folic acid deficiency, and hypoalbuminemia are at risk for MTX-induced pancytopenia, which was observed in our case [5]. As a presenting characteristic of MTX toxicity, our patient had mucositis and myelosuppression. The patient's myelosuppression was more likely caused by an inadvertent MTX dosage and by old age.

The main contributory cause of the toxicity is an inadvertent MTX dose. Patients, family members, or relatives who are adjusting the dosing of such medications must be avoided at all costs. Patients should be properly counseled on not taking the medications without seeing a doctor and not combining them with other drugs without a doctor's permission. The general population should be made aware of high-risk medications like this. In a country like India, such drugs should not be sold without a prescription. Renal functions that are already impaired can go unnoticed, and renal clearance dysfunction may have played a role in MTX toxicity.

Blood checks are required every 12 weeks if the patient has been on the drug for more than six months [6]. MTX is otherwise safe and successful in cases of psoriasis, but close monitoring is needed. This case shows the importance of clear and consistent contact with patients while a medication like methotrexate is administered, because although it has been proven to be a good drug, side effects will manifest and can be life-threatening if they arise. In Figures 1-4, we can see hemorrhagic patches all over the body parts and mouth ulceration due to methotrexate toxicity.

Kivity et al. studied 28 patients presenting with low-dose methotrexate overdose and found pancytopenia to be the most common presenting feature (78%). Acute renal dysfunction, hypoalbuminemia, concurrent use of medications believed to interfere with methotrexate, and dosage errors were all risk factors. However, the methotrexate levels did not correlate with the degree of neutropenia or thrombocytopenia, and they did not even differ in patients who died of toxicity or survived it. All the patients mostly died as a result of sepsis resulting from myelosuppression [7].

Calvo Romero discovered that pancytopenia caused by low-dose methotrexate treatment is more likely to develop in the presence of renal dysfunction. [8] Shaikh et al. studied 40 cases of methotrexate-receiving patients who had developed myelosuppression. The most common factors responsible for the development of myelosuppression as reported by them were inadequate or no use of folic acid supplements, concurrent medication, and low renal function. They also concluded that myelosuppression can occur at any time during treatment, and the severity of the disease was not dependent on the dosage of methotrexate [9].

This patient had been prescribed methotrexate 7.5 mg to be taken once a week, which was apparent from the prescriptions that she had with her. However, she had been given it daily for the past 15 days by one of the relatives before landing up in a critical state. Singh and Handa reported a similar case where toxicity occurred as a result of a prescription error, which was similar to this one where the patient misunderstood the frequency of doses that she had to take [10]. Grissinger reported one such similar case where the patient took the drug daily instead of weekly [11].

This case makes the need for proper and repeated counseling of patients regarding the dosage of drugs at all levels of contact with health personnel very clear. The manifestations of methotrexate overdose need to be carefully watched for early diagnosis and treated aggressively if present. We describe a fatal case of methotrexate overdose in a patient with psoriasis who was treated after an accidental overdose.

## Conclusions

Pharmacists and medical workers should be extra careful while dispensing drugs. They should crosscheck with a doctor if they have any doubts about it.

Relatives who administer drugs should be completely knowledgeable about how to, how much and when to give and what possible side effects should be observed. These obstacles should be avoided for patients' safety.
